# Assessment of Left Ventricular Systolic Dysfunction in Patients Undergoing Primary Percutaneous Coronary Intervention for Anterior Wall ST-Segment Elevation Myocardial Infarction After 48 Hours in the Cardiac Emergency

**DOI:** 10.7759/cureus.113099

**Published:** 2026-07-21

**Authors:** Ahmad Raees, Areeha Raees, Ambreen T Chattha, Iannis Flogaitis

**Affiliations:** 1 Department of Cardiology, Faisalabad Institute of Cardiology, Faisalabad, PAK; 2 Department of Medicine, Aziz Fatimah Medical and Dental College, Faisalabad, PAK; 3 Department of Forensic Medicine, Abwa Medical College, Faisalabad, PAK; 4 Department of Cardiology, The Hillingdon Hospitals NHS Foundation Trust, London, GBR

**Keywords:** 2d echocardiography, anterior wall stemi, color doppler assessment, left ventricular ejection fraction, percutaneous coronary intervention

## Abstract

Background

This study aimed to compare left ventricular ejection fraction (LVEF) before and 48 hours after primary percutaneous coronary intervention (PPCI) using paired assessments in patients presenting with anterior wall ST-segment elevation myocardial infarction (STEMI).

Methodology

This prospective, observational, before-and-after study was conducted in the Department of Cardiology, Faisalabad Institute of Cardiology (FIC), Faisalabad, from June 12, 2021, to December 11, 2021. A total of 50 patients of either gender aged 18 to 50 years with acute anterior wall STEMI undergoing PPCI in the emergency were included. Patients with coronary artery bypass graft, atrial fibrillation, atrial flutter or complete heart block, cardiogenic shock, and valvular heart disease were excluded. PPCI with stenting was performed using the standard technique on all eligible consenting patients as per the department’s protocol. All data were collected on a performa by the primary investigator. Two-dimensional echocardiography with color Doppler was performed at baseline and at 48 hours after PPCI, and LVEF was estimated.

Results

The age range in this study was from 18 to 50 years, with a mean age of 38.74 ± 7.50 years. The majority of the patients (35, 70.0%) were between 36 and 50 years of age. Out of the 50 patients, 27 (54.0%) were male, and 23 (46.0%) were female, with a ratio of 1.2:1. Left ventricular systolic function improved from a baseline of 39.46 ± 4.63% to 56.36 ± 4.68% after PPCI performed in emergency in patients with anterior wall STEMI (p = 0.001).

Conclusions

Left ventricular systolic function (LVEF) is improved 48 hours after PPCI in patients with anterior wall STEMI in cardiac emergency.

## Introduction

Acute myocardial infarction (AMI) remains a leading cause of heart failure despite significant treatment advancements in recent years [1]. The occurrence of new-onset heart failure following AMI is associated with an unfavorable prognosis and increased in-hospital mortality. Numerous studies have shown that timely administration of primary percutaneous coronary intervention (PPCI) in patients with ST-segment elevation myocardial infarction (STEMI) significantly decreases both mortality and morbidity [2].

STEMI is a clinical entity defined by evidence of myocardial ischemia manifested through characteristic symptoms, persistent ST-segment elevation on electrocardiography, and the subsequent rise of cardiac biomarkers indicating myocardial necrosis. Ischemic heart disease remains the leading cause of mortality worldwide, with its prevalence continuing to rise globally. PPCI has become the treatment of choice for patients presenting with STEMI. However, functional recovery following PPCI is variable, and certain patients experience persistent or progressive ventricular dysfunction [3].

Left ventricular ejection fraction (LVEF) constitutes a critical clinical determinant strongly associated with an increased risk of developing congestive heart failure and decreased long-term survival. Patients with asymptomatic ventricular dysfunction exhibit mortality rates nearly five times higher than those of the general population. Assessment of regional left ventricular function serves as a valuable diagnostic approach for detecting myocardial segments with compromised coronary perfusion and impaired contractility [4,5]. PCI serves as an effective reperfusion strategy that enhances both global and regional myocardial function, contributing significantly to improved clinical outcomes and quality of life.

PPCI in patients with STEMI can substantially reduce infarct size and preserve LVEF. However, functional recovery is not universally achieved, as evidence suggests that approximately 4.7-8.6% of patients exhibit a decline in cardiac function despite technically successful PPCI. Additionally, between 10% and 30% of patients undergoing PCI demonstrate left ventricular dysfunction [6]. Remmelink et al. reported that systolic function improved from a baseline of 40% ± 17% to 54% ± 15% following emergency PPCI in anterior STEMI patients (p = 0.001) [7].

Currently, no local data exists regarding the prevalence of systolic dysfunction in patients undergoing PPCI in the cardiac emergency setting following anterior wall STEMI. Early diagnosis and management of left ventricular dysfunction in anterior wall STEMI patients can substantially reduce morbidity and mortality. Accordingly, this study was designed to compare LVEF in patients with anterior STEMI at baseline and at 48 hours following emergency PPCI.

## Materials and methods

This prospective, observational, before-and-after study was conducted in the Department of Cardiology, Faisalabad Institute of Cardiology (FIC), Faisalabad, from June 12, 2021, to December 11, 2021. All patients of either gender aged 18 to 50 years with acute anterior wall STEMI, as per the operational definition, undergoing PPCI in the emergency department were included in the study. A sample size of 50 patients was calculated using the Open Epi sample size calculator, with 80% power, a 5% significance level, and expected LVEF values of 40% ± 17% at baseline and 54% ± 15% after PPCI. Patients with a prior history of MI, coronary artery bypass graft, PCI, pericardial disease, atrial fibrillation, atrial flutter or complete heart block, cardiogenic shock, valvular heart disease, and with very poor control of diabetes were excluded.

After obtaining approval from the hospital’s Ethical Review Committee (approval number: 10/DME/FIC/FSD), patients were enrolled through emergency, and informed consent was obtained. A detailed clinical history, demographic profile, and physical examination findings were documented, with particular emphasis on coronary risk factors such as smoking, dyslipidemia, hypertension, diabetes mellitus, family history of ischemic heart disease, and previous MI. Routine biochemical investigations were performed, and pharmacotherapy was initiated following departmental protocols. All eligible and consenting patients underwent PPCI with stent placement using standard procedural techniques. Data for each participant were recorded on a structured proforma by the principal investigator. Duration of chest pain till admission to the emergency, HbA1c level, and pre-PCI Thrombolysis In Myocardial Infarction (TIMI) flow seen during PPCI were also noted.

LVEF was assessed by transthoracic echocardiography at two predefined time points: before PPCI (baseline) and 48 hours after the procedure. All examinations were performed using the same echocardiography system by experienced cardiologists according to standard institutional protocols. LVEF was measured using the modified Simpson's biplane method whenever image quality permitted. Because measurements were performed as part of routine clinical care, formal assessment of interobserver and intraobserver variability was not performed.

All data were analyzed using SPSS version 20 (IBM Corp., Armonk, NY, USA). Qualitative data including gender, duration of chest pain, and TIMI flow were analyzed using frequency and percentage, and quantitative data including age, HbA1c, and LVEF at baseline and after PPCI were analyzed using mean ± standard deviation (SD).

Mean LVEF at baseline was compared with LVEF measured 48 hours after PPCI using a paired-samples t-test, as both measurements were obtained from the same participants. A p-value ≤0.05 was considered statistically significant. Effect modifiers including age, gender, and duration of chest pain were evaluated by stratification.

Stratified analyses according to age, gender, and duration of chest pain were prespecified in the study protocol to explore potential differences in post-PPCI LVEF. However, the sample size was calculated based on the primary outcome of change in LVEF and was not specifically powered to detect statistically significant differences within these subgroups.

## Results

**Figure 1:**
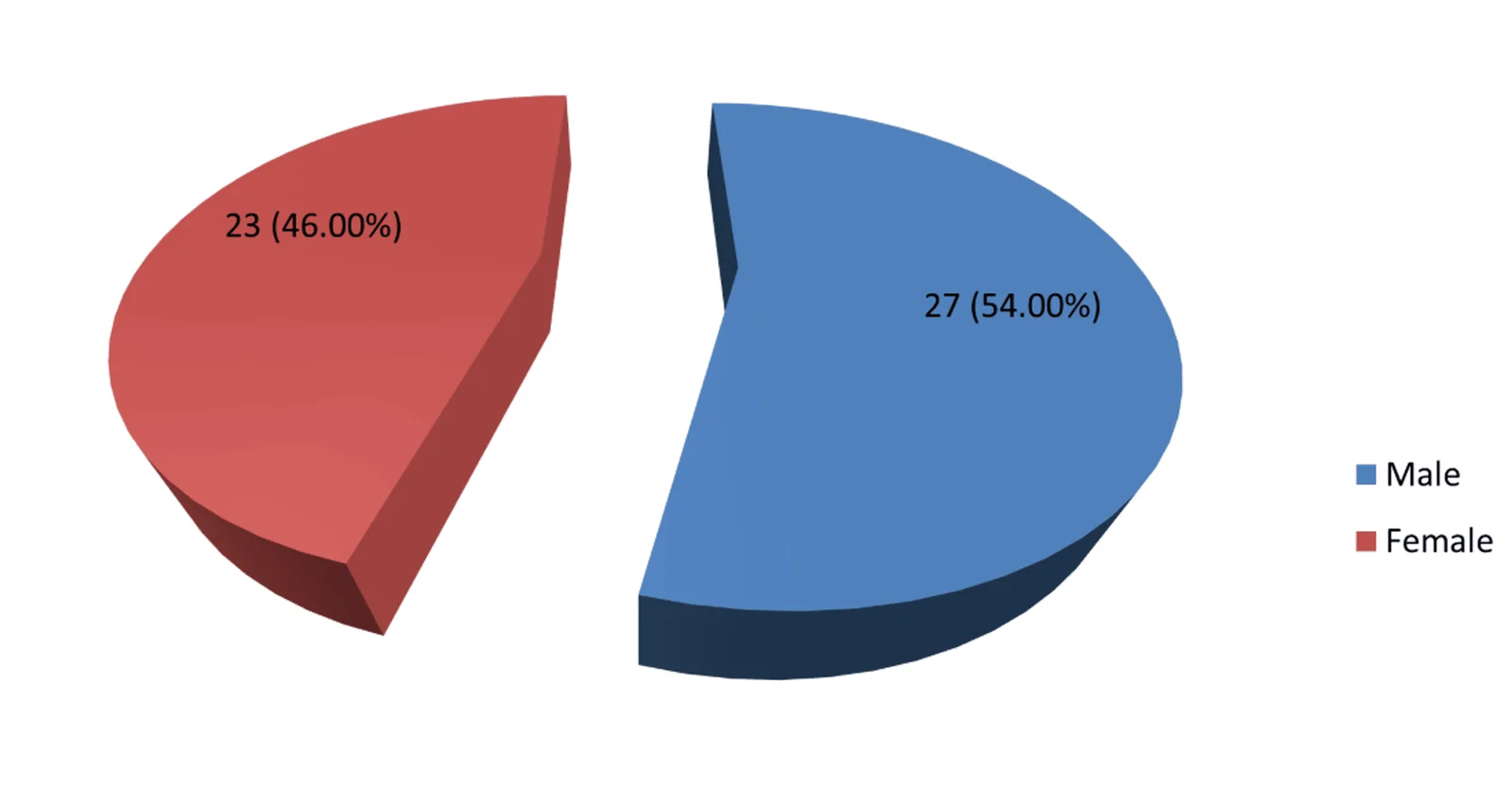
Distribution of patients according to gender (n = 50).

The age range in this study was from 18 to 50 years, with a mean age of 38.74 ± 7.50 years. The majority of the patients (35, 70.0%) were between 36 and 50 years of age. Of the 50 patients, 27 (54.0%) were male, and 23 (46.0%) were female, with a ratio of 1.2:1 (Figure 1).

The age, gender, and duration of chest pain of the study participants are shown in Table 1.

**Table 1:** Distribution of patients according to age, gender, and duration of chest pain (n = 50).

Variable		Number of patients	%
Age (years)	18–35	15	30.0
	36–50	35	70.0
Gender	Male	27	54.0
	Female	23	46.0
Duration (hours)	≤6 hours	28	56.0
	>6 hours	22	44.0
Total		50	100.0

The mean duration of chest pain was 5.94 ± 2.61 hours. The mean HbA1c was 6.34% ± 2.12%. TIMI flow grade was 0 in 17 (34.0%), grade 1 in 21 (42.0%), and grade 2 in 12 (24.0%) patients, as illustrated in Table 2.

**Table 2:** Distribution of patients according to pre-PCI, TIMI flow grade. PCI = percutaneous coronary intervention; TIMI = Thrombolysis In Myocardial Infarction

TIMI flow	Grade 0	Grade I	Grade II
Patient proportion	17 (34.0%)	21 (42%)	12 (24%)

Pre-PCI TIMI flow grades were classified as follows: TIMI 0 refers to the absence of any antegrade flow beyond coronary occlusion. TIMI 1 flow is faint antegrade coronary flow beyond the occlusion, although filling of the distal coronary bed is incomplete. TIMI 2 flow is delayed or sluggish antegrade flow with complete filling of the distal territory.

The mean LVEF improved significantly from 39.46% ± 4.63% at baseline to 56.36% ± 4.68% at 48 hours after PPCI. The mean improvement in LVEF was 16.90 percentage points (95% confidence interval (CI) = 15.36-18.44). This difference was statistically significant on a paired-samples t-test (t(49) = 22.10, p < 0.001), as shown in Table 3.

**Table 3:** Comparison of baseline and 48-hour LVEF using a paired-samples t-test. LVEF = left ventricular ejection fraction; SD = standard deviation; CI = confidence interval

Variable	Baseline, mean ± SD	48-hour, mean ± SD	Mean difference (95% CI)	t (df)	P-value
LVEF (%)	39.46 ± 4.63	56.36 ± 4.68	16.90 (15.36 to 18.44)	22.10 (49)	<0.001

The stratification of LVEF at 48 hours of PPCI with respect to age, gender, and duration of chest pain is shown in Table 4.

**Table 4:** Stratification of LVEF 48 hours after PPCI with respect to age groups, gender, and duration of chest pain using independent-sample t-test. LVEF = left ventricular ejection fraction; PPCI = primary percutaneous coronary intervention; SD = standard deviation

		LVEF		P-value
		Mean	SD	
Age (years)	18–35	58.0	4.78	0.105
	36–50	55.66	4.52	
Gender	Male	56.85	5.28	0.426
	Female	55.78	3.90	
Duration (hours)	≤6	55.75	4.39	0.303
	>6	57.14	5.01	

## Discussion

The present study demonstrated a significant improvement in left ventricular systolic function following PPCI in patients presenting with anterior wall STEMI. LVEF improved substantially from 39.46% ± 4.63% at baseline to 56.36% ± 4.68% at 48 hours after PPCI, representing a mean absolute improvement of 16.90 percentage points (95% CI = 15.36-18.44), indicating a clinically meaningful recovery of left ventricular systolic function.

This improvement aligns with current evidence supporting PPCI as the gold standard reperfusion strategy for AMI [8]. The rapid restoration of coronary flow achieved through PPCI limits myocardial necrosis and preserves ventricular function, which is critical in determining both short-term survival and long-term morbidity [9]. The baseline LVEF of approximately 39% in our cohort reflects substantial myocardial injury typical of anterior wall STEMI, as the left anterior descending coronary artery supplies the anteroseptal and anterolateral walls, making anterior infarcts particularly destructive to left ventricular performance [10].

The recovery observed within 48 hours likely reflects myocardial stunning, a transient myocardial dysfunction despite restoration of coronary perfusion [11]. This mechanical dysfunction gradually resolves as the ischemic myocardium recovers metabolic function. Studies have consistently demonstrated that early intervention minimizes the extent of irreversible myocardial damage, allowing greater potential for functional recovery [12]. Following PPCI, improved microvascular perfusion and reduced reperfusion injury collectively contribute to this functional improvement [8].

Our analysis reveals that patients presenting within six hours of symptom onset achieved a mean LVEF of 55.75% ± 4.39% compared to 57.14% ± 5.01% in those presenting beyond six hours, though this difference was not statistically significant (p = 0.303). While the lack of statistical significance may reflect the relatively small sample size and limited heterogeneity in reperfusion timing, it also suggests that salvageable myocardium remains viable even beyond the traditional six-hour window. However, the apparent trend toward slightly better outcomes in the >6 hours group should not be over-interpreted, as this likely represents selection bias or the “open artery hypothesis,” where some myocardium remains viable longer than the classical timeframe suggests [13].

Earlier guidelines emphasized the “time is muscle” principle, but contemporary evidence supports extending mechanical reperfusion opportunities to patients presenting within 12-24 hours of symptom onset, particularly in anterior STEMI where the myocardial territory at risk is substantial. Recent evidence supports PPCI regardless of symptom duration in appropriately selected patients, as delayed presentation does not preclude clinical benefit [14].

Stratification by age groups demonstrated that younger patients (18-35 years) achieved slightly higher mean LVEF (58.0% ± 4.78%) compared to those aged 36-50 years (55.66% ± 4.52%), though this difference lacked statistical significance (p = 0.105). This trend likely reflects better baseline cardiac reserve and fewer comorbidities in younger populations, which facilitate more complete functional recovery [12]. However, the modest absolute difference (2.34%) and lack of statistical significance suggest that age alone should not preclude aggressive intervention in older patients, as substantial functional recovery remains achievable across age groups.

Regarding gender distribution, our cohort comprised 54.0% males and 46.0% females (ratio = 1.2:1), with post-PPCI LVEF being slightly higher in males (56.85% ± 5.28%) compared to females (55.78% ± 3.90%) (p = 0.426). This non-significant difference contrasts with some literature suggesting worse outcomes in women with acute coronary syndromes. However, emerging evidence indicates that differences in outcomes between genders may reflect presentation delays, atypical symptomatology leading to diagnostic delays, and fewer invasive procedures rather than intrinsic biological differences in myocardial recovery potential [14].

The distribution of pre-PCI TIMI flow grades in our study was as follows: grade 0 in 34.0% of patients, grade 1 in 42.0%, and grade 2 in 24.0%. Although post-procedural TIMI flow was not evaluated in this study, a significant improvement in LVEF was observed across the cohort following PPCI [9]. The relationship between TIMI flow grade and LVEF recovery has been well-documented, with higher TIMI flow grade associated with superior functional outcomes compared to lower grades [8-10]. Importantly, despite 34% of patients presenting with complete coronary occlusion (pre-procedural TIMI 0 flow), a significant improvement in LVEF was observed after PPCI, supporting the beneficial effect of early reperfusion in restoring ventricular systolic function.

The mean HbA1c of 6.34 ± 2.12% in our population indicates that approximately one-third of patients had glycemic dysregulation. Diabetes mellitus and impaired glycemic control are established risk factors for larger infarct size and impaired myocardial recovery [10]. The relatively modest elevation in mean HbA1c in our cohort may have contributed to the favorable functional recovery observed. However, glycemic control in the acute setting and subsequent chronic management are crucial determinants of long-term outcomes and should be systematically optimized in all post-STEMI patients [12].

The strengths of this study include its prospective observational design, standardized assessment of LVEF before and 48 hours after PPCI, and the evaluation of ventricular systolic function using paired measurements within the same patients. These features allowed objective assessment of the early changes in left ventricular function following successful reperfusion.

Although a significant improvement in LVEF was observed 48 hours after PPCI, the findings should be interpreted in the context of the study design, as without a comparison group, the study cannot establish a causal relationship between PPCI and the observed improvement in left ventricular function. This study represents a single-center experience with a modest sample size (n = 50), which limits generalizability and the power to detect significant differences in stratified analyses. Furthermore, although subgroup analyses according to age, gender, and duration of chest pain were prespecified, the study was not adequately powered to detect differences within these subgroups. Consequently, these findings should be regarded as exploratory and interpreted with caution.

The relatively short follow-up period of 48 hours captures acute myocardial stunning rather than long-term remodeling and definitive functional recovery [11]. Longer follow-up periods, ideally extending to three to six months, would better characterize sustained functional improvement and identify patients at risk for progressive ventricular dilatation and heart failure. Additionally, detailed analysis of procedural factors such as mechanical circulatory support and deployment of adjunctive devices would provide mechanistic insights into determinants of functional recovery [15].

## Conclusions

In this prospective observational study, patients with anterior wall STEMI demonstrated a significant improvement in LVEF 48 hours after undergoing PPCI. These findings suggest that early recovery of left ventricular systolic function can be detected following successful reperfusion. Assessment of LVEF at 48 hours may provide useful information regarding early ventricular functional recovery after PPCI. However, because of the observational study design, absence of a comparison group, and short follow-up period, these findings should be interpreted with caution and should not be considered evidence of a causal effect. Larger multicenter prospective studies with longer follow-up are needed to confirm these findings and evaluate their relationship with long-term clinical outcomes.
